# Single Cell RNA‐Seq Identifies Cell Subpopulations Contributing to Idiopathic Pulmonary Fibrosis in Humans

**DOI:** 10.1111/jcmm.70402

**Published:** 2025-02-10

**Authors:** Tangjuan Zhang, Zhichao Hou, Zheng Ding, Peng Wang, Xue Pan, Xiangnan Li

**Affiliations:** ^1^ Department of Emergency The First Affiliated Hospital of Zhengzhou University Zhengzhou China; ^2^ Department of Thoracic Surgery The First Affiliated Hospital of Zhengzhou University Zhengzhou China; ^3^ School of Nursing and Health Zhengzhou University Zhengzhou China

**Keywords:** cell–cell interaction, end‐stage lung disease, fibroblast, idiopathic pulmonary fibrosis, interstitial lung disease, macrophage, scRNA‐seq, T and B cells

## Abstract

The cell populations, particularly subpopulations, involved in the onset and progression of idiopathic pulmonary fibrosis (IPF) remain incompletely understood. This study employed single‐cell RNA‐seq to identify cell populations and subpopulations with significantly altered proportions in the lungs of patients with IPF. In IPF lungs, endothelial cell proportions were significantly increased, while alveolar epithelial cell proportions were markedly decreased. Among the three identified fibroblast subpopulations, the proportion of myofibroblasts was significantly increased, while the proportions of the other two fibroblast subtypes were reduced. Similarly, within the three macrophage subpopulations, the macrophage_SPP1 subpopulation, localised to fibroblastic foci, showed a significant increase in proportion, while the alveolar macrophage subpopulation was significantly reduced. Trajectory analysis revealed that fibroblasts in IPF lungs could differentiate into myofibroblasts, and alveolar macrophages could transition into the macrophage_SPP1 subpopulation. Among T‐cell subpopulations, only the CD4 T_FOXP3 subpopulation exhibited a significant change, whereas all four B‐cell subpopulations showed significant proportional shifts. These findings provide a comprehensive view of the cellular alterations contributing to IPF pathogenesis. Extensive interactions among various cell populations and subpopulations were identified. The proportions of various cell populations and subpopulations in IPF lungs, including endothelial cells, fibroblasts, macrophages and B cells, were significantly altered. Further in‐depth investigation into the roles of cell subpopulations with significantly altered proportions in the onset and progression of IPF will provide valuable insights into the pathological mechanisms underlying the disease. This understanding could facilitate the development of novel therapeutic strategies and medications for IPF treatment.

## 1 Introduction

Idiopathic pulmonary fibrosis (IPF) is a chronic, progressive, fibrotic interstitial lung disease of unknown aetiology, characterised by limited treatment options and a high mortality rate [1, 2, 3, 4, 5]. Despite its variable and unpredictable course, the median survival time following diagnosis is only 2–4 years [6]. Epidemiological studies report an incidence of 2–30 cases per 100,000 person‐years, depending on geographic region, with overall incidence increasing over time [7, 8, 9, 10]. The prevalence of IPF rises dramatically with age, reaching an estimated 400 cases per 100,000 individuals in patients aged 65 years or older [11]. Currently, only a few medications, such as nintedanib and pirfenidone, are recommended for the treatment of IPF [12]. This limitation may be due, in part, to the incomplete understanding of the underlying mechanisms of fibrosis in IPF [5].

It is well established that various cell types, including epithelial cells, endothelial cells, fibroblasts, macrophages and T cells, contribute to the pathological process of IPF [3]. However, the subpopulations of these cells and their altered proportions in the lungs of IPF patients remain incompletely understood. Furthermore, cell–cell interactions in the context of IPF pathophysiology are even less explored. Single‐cell RNA sequencing (scRNA‐seq) is a powerful tool for addressing these questions [13, 14]. Several scRNA‐seq studies have implicated epithelial cells, mesenchymal cells, myofibroblasts, endothelial cells and macrophages in the development of pulmonary fibrosis in IPF [15, 16, 17]. However, these studies have not conducted in‐depth analyses of changes in subpopulation proportions, potential differentiation or transformation among subpopulations, and the intricate cell–cell interactions that may drive disease progression.

In contrast, the current research provides an in‐depth analysis of cell subpopulations and their proportional changes, focusing on fibroblasts, macrophages, T cells and B cells. This study also explores potential differentiation pathways among fibroblast subpopulations and macrophage subpopulations. Additionally, detailed investigations into cell–cell interactions have been conducted, encompassing interactions among various cell types and their subpopulations. These findings offer invaluable insights for future research aimed at identifying specific cell populations or subpopulations that contribute to the onset and progression of IPF.

## 2 Material and Methods

### 2.1 Sample Collection, Processing, Library Preparation and Sequencing

Clinical samples were obtained from patients diagnosed with end‐stage IPF who underwent lung transplantation between 2021 and 2022. End‐stage IPF is diagnosed in the absence of secondary causes and is defined as primary fibrosing interstitial lung disease meeting at least one of the following criteria: (1) Carbon monoxide diffusion capacity (DLCO): Less than 39% of the predicted value. (2) Forced vital capacity (FVC): A decrease exceeding 10% within 6 months. (3) Six‐minute walk test (6‐MWT): Blood oxygen saturation (SpO_2_) drops below 88% during the test. (4) High‐resolution CT (HRCT): Evidence of disease progression. Five IPF patients meeting these criteria were selected for the study. Detailed pathological information is provided in Table 1, and corresponding CT diagnostic images are shown in Figure S1. Sample collection and testing were approved by the ethics committee of the First Affiliated Hospital of Zhengzhou University and adhered to national ethical standards for human research. The control group included five healthy donors recruited through the National Organ Donation Platform, with all personal information strictly protected. Both preoperative CT scans and postoperative pathological examinations confirmed the absence of lung disease in these donors, verifying that their lungs were normal.

**TABLE 1:** Baseline characteristics and lung function of IPF patients.

Patient ID	Gender	Age (years)	Medical history (years)	Smoking	6‐MWT	Lung function	Note
IZPX2	Male	60	4	No	360 m; SpO_2_ < 75%	FEV1 1.22L, 42.4%; FVC 1.36L, 38.1%; DLCO(SB)12.6%; DLCO/VA 43.3%	No
ZXWH‐Z	Male	69	6	No	SpO_2_ < 88%	FVC decreased by > 10% within 6 months; DLCO decreased by > 15% within 6 months	Resting state: SpO_2_ 90%–93% with oxygen 6 L/min; Minimal physical activity: severe dyspnoea, SpO_2_ < 80%
LYF‐01	Female	53	4	No	383 m; SpO_2_ < 88%	FVC 1.69L, 57.9%; FEV1/FVC 114.4%; DLCO(SB) 34.3%	No
XD‐01	Female	60	10	No	UA	FVC 0.856L, 36.9%; FEV1 0.781L, 45.2%; FEV1/FVC 91.2%	Unable to wean off oxygen inhalation; 6‐MWT: Unable to complete
JBX‐Z	Male	65	3	No	UA	UA	High‐flow nasal oxygen inhalation (FiO_2_ 70%–100%); Unable to get out of bed; Pulse oxygen drop to about 70% with coughing; Critical condition

Abbreviations: 6‐MWT, six‐minute walk test; DLCO (SB), single‐breath diffusing capacity of the lung for CO; FEV1, forced expiratory volume in one second; FiO_2_, fraction of inspired oxygen; FVC, forced vital capacity; SpO_2_, oxygen saturation; VA, alveolar volume.

For the analysis, lung tissue samples from IPF patients were selected from regions exhibiting severe lesions, as identified by CT scans, and preserved in MACS Tissue Storage Solution (Miltenyi Biotec) until further processing. The tissue preparation involved the following steps: Samples were washed with phosphate‐buffered saline (PBS) and minced into small pieces (approximately 1 mm^3^) on ice. The minced tissue was enzymatically digested with a solution containing 1 mg/mL collagenase I (Gibco), 1 mg/mL collagenase II, 60 U/mL hyaluronidase (Sigma), 10 U/mL Liberase (Roche) and 0.02 mg/mL DNase I (Roche) for 90 min at 37°C with agitation. Following digestion, the samples were filtered through 100‐ and 40‐μm cell strainers and centrifuged at 300 *g* for 5 min. The supernatant was removed, and the pelleted cells were suspended in red blood cell lysis buffer (Gibco) to lyse red blood cells. The cells were washed with DPBS‐containing sample buffer, and the cell pellets were resuspended in DPBS‐containing sample buffer. The cells were then stained and counted for subsequent analysis.

The BD Rhapsody system was employed to capture transcriptomic information from single cells isolated from IPF patients and normal controls. The workflow consisted of the following steps: Single‐cell capture was achieved by distributing a single‐cell suspension across over 200,000 microwells through a limited dilution approach. Beads with oligonucleotide barcodes were added to saturation, ensuring that each bead was paired with a cell in a microwell. Cells were lysed within the microwells, allowing mRNA molecules to hybridise to barcoded capture oligos on the beads. Beads were then collected into a single tube for reverse transcription and ExoI digestion. During cDNA synthesis, each cDNA molecule was tagged on the 5′ end (the 3′ end of an mRNA transcript) with a unique molecular identifier (UMI) and a cell barcode indicating its cell of origin. Whole transcriptome libraries were prepared using the BD Rhapsody single‐cell whole‐transcriptome amplification (WTA) workflow, which included random priming and extension (RPE), RPE amplification PCR and WTA index PCR. The libraries were quantified using a High Sensitivity D1000 ScreenTape (Agilent) and High Sensitivity D1000 Reagents (Agilent) on a 4150 TapeStation System (Agilent), as well as the Qubit High Sensitivity DNA assay (Thermo Fisher Scientific). Sequencing was performed on an Illumina sequencer (Illumina, San Diego, CA) using a 150‐bp paired‐end run.

### 2.2 Data Analysis and Statistics

After sequencing, the expression matrix was generated using Cellranger software (v7.2.0, https://www.10xgenomics.com/software). The matrix was then imported into R (v4.1.1), and cell populations or subpopulations were identified using the Seurat package (v5.1.0). This process involved a series of steps, including quality control, principal component analysis (RunPCA), dimensionality reduction (RunTSNE or RunUMAP) and clustering (FindClusters) [18, 19, 20]. Cell population and subpopulation counts were extracted, and proportional plots were created using the ggplot2 package in R (v4.1.1). Statistical comparisons between two groups were performed using Student's *t*‐test in R, with significance defined as a *p*‐value less than 0.05. No comparisons among multiple groups were included in this study. Cell–cell interactions were analysed using CellphoneDB (v4.1.0) according to the software's guidelines [21, 22]. Single‐cell trajectory analysis was conducted in R using Monocle3 (v1.2.7) [23, 24]. Specifically, Seurat objects for different cell types were extracted from the integrated Seurat object and converted into Monocle‐compatible datasets. Cells were clustered using the cluster_cells function with a resolution parameter of 1e‐3. Developmental trajectories were constructed using the learn_graph function, which incorporated partitions to delineate distinct cell groupings. Cells were then ordered along the inferred trajectory using UMAP‐based dimensionality reduction for visualisation and trajectory analysis. All code used in this research will be made available upon request.

## 3 Results

### 3.1 Single Cell RNA‐Seq Reveals Human Lung Cell Populations and Changes in the Lungs of Patients With Idiopathic Pulmonary Fibrosis

**FIGURE 1:**
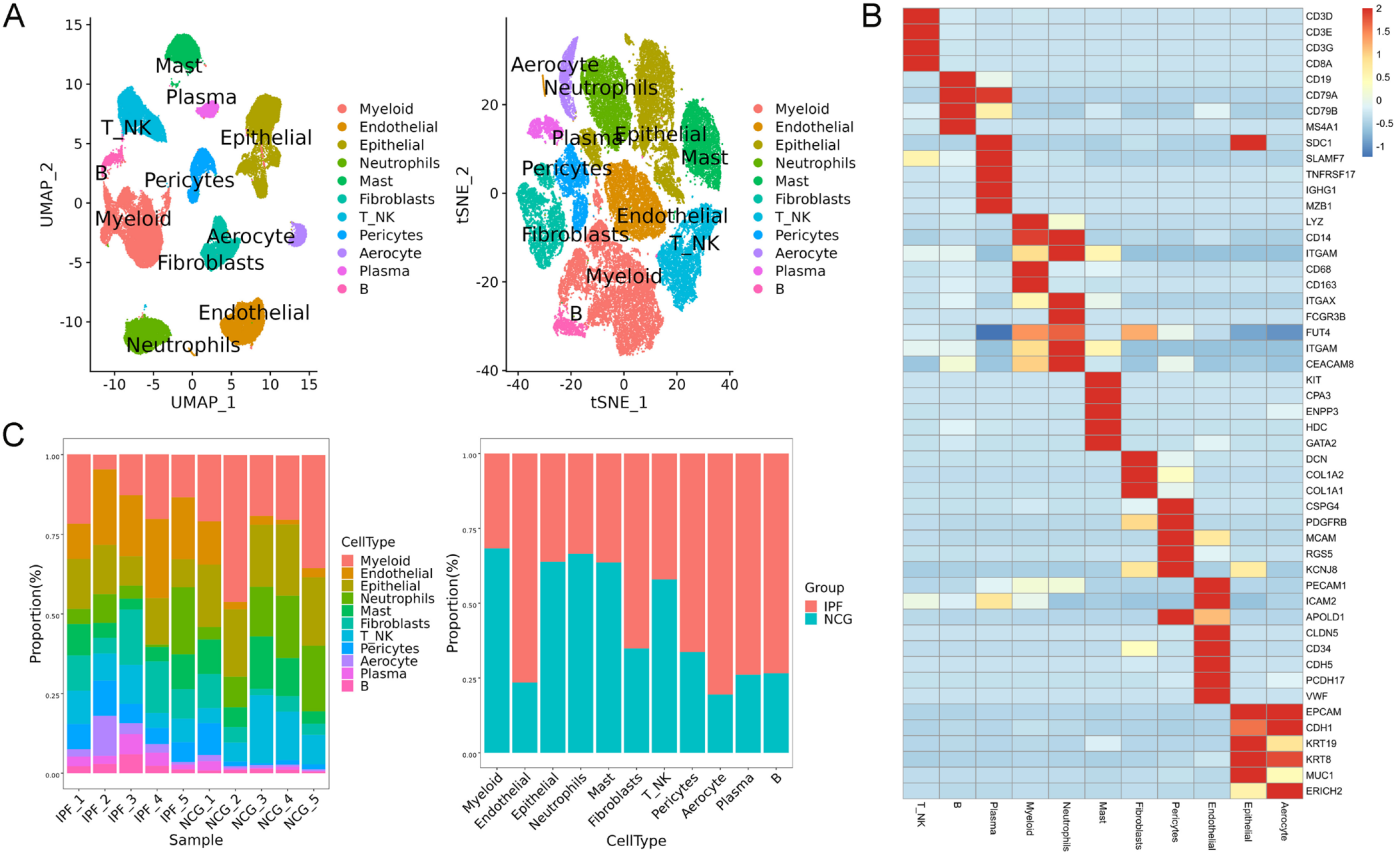
Single‐cell RNA‐seq was used to identify cell types in human lungs and their alterations in the lungs of patients with idiopathic pulmonary fibrosis (IPF). The analysis included five IPF lung samples and five normal control lung samples. The single‐cell RNA‐seq data were integrated, and batch effects were effectively corrected. Cell populations and subpopulations, along with their respective marker genes, were identified using the Seurat package. (A) Cell types in human lungs identified by single‐cell RNA‐seq. (B) Marker genes expressed in cell populations identified by single‐cell RNA‐seq. (C) Changes in the proportions of different cell populations in the lungs of IPF patients compared to normal human lungs.

Idiopathic pulmonary fibrosis (IPF) is a chronic, progressive, fibrotic interstitial lung disease of unknown cause, characterised by high mortality and limited treatment options. Alterations in cell proportions and cell–cell interactions are thought to play a critical role in the pathogenesis and progression of IPF [3]. Understanding these changes could provide crucial insights into the pathological mechanisms of IPF and aid in the development of more effective treatment strategies and therapeutics. This study aimed to investigate these changes by performing scRNA‐seq on lung tissues obtained from both healthy individuals and patients with IPF who underwent lung transplantation due to respiratory failure. The experimental workflow is outlined in Figure S2. Approximately 60,000 cells were sequenced from the lungs of five IPF patients and five healthy controls. To provide a comprehensive understanding of the scRNA‐seq data, the analysis first identified major cell types present in the lung tissues, followed by a detailed examination of their subpopulations. Common cell populations were defined based on established marker molecules, including endothelial cells, epithelial cells, neutrophils, mast cells, fibroblasts, pericytes, aerocytes, B cells, myeloid‐derived cells and natural killer T (NKT) cells (Figure 1A,B). The marker genes were selected based on their expression profiles across all lung samples, encompassing both IPF patients and healthy controls (Figure 1B). Compared to normal controls, the proportions of endothelial cells, fibroblasts, pericytes, aerocytes and plasma/B cells were markedly increased in IPF lungs, with proportions exceeding 50% (Figure 1C). Conversely, the proportions of myeloid cells, epithelial cells, neutrophils, mast cells and NKT cells were significantly decreased, with proportions falling below 50% (Figure 1C). These changes in cell proportions are likely associated with the pathophysiology of IPF, highlighting potential cellular contributors to disease onset and progression.

### 3.2 Single Cell RNA‐Seq Identifies Fibroblast Subpopulations and Their Proportion Changes in the Lungs of IPF Patients

**FIGURE 2:**
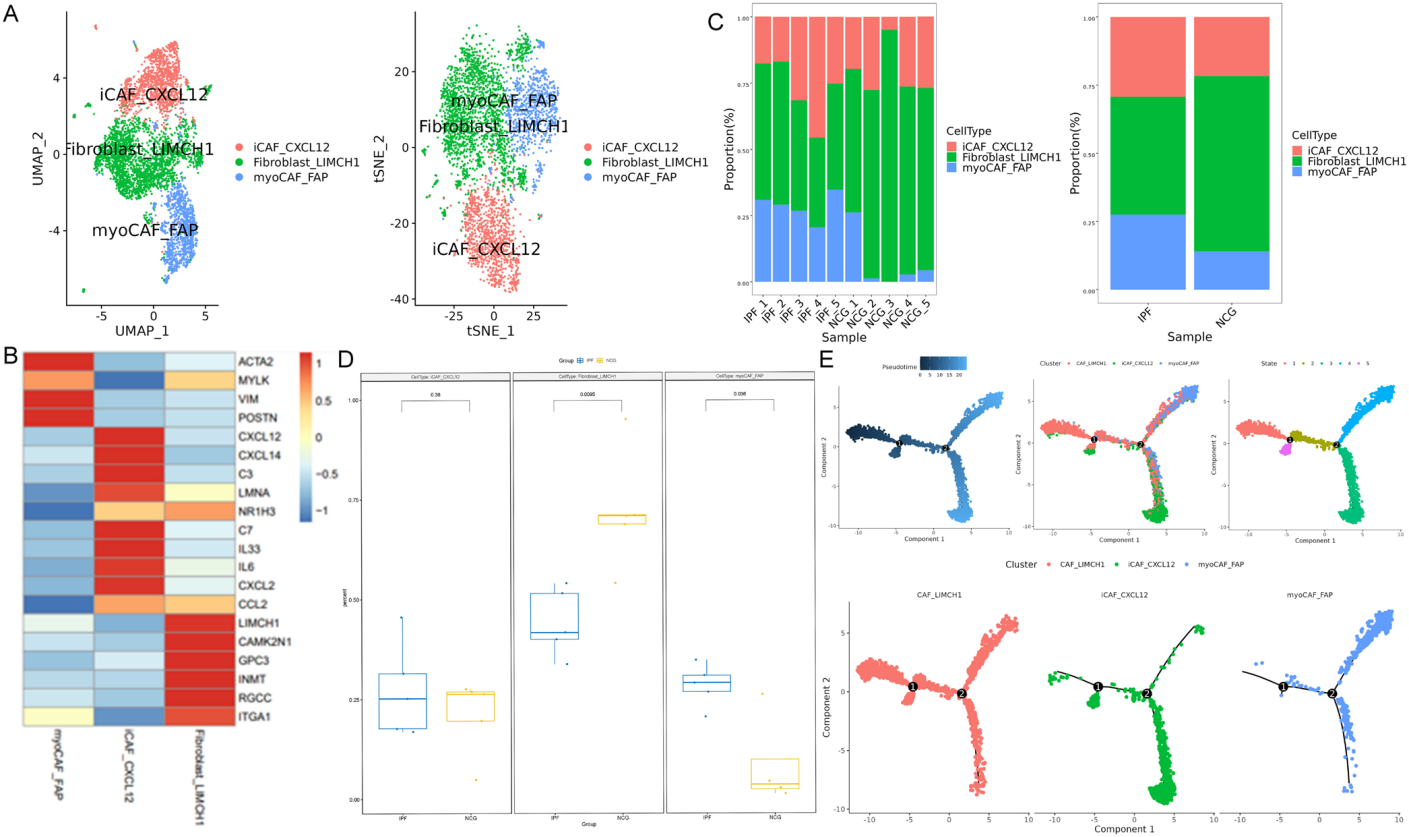
Fibroblast subpopulations identified by single‐cell RNA‐seq and their alterations in the lungs of patients with idiopathic pulmonary fibrosis (IPF). Fibroblast cells were extracted from the complete Seurat object and processed using the standard Seurat pipeline to identify and characterise fibroblast subpopulations. (A) Single‐cell RNA‐seq revealed three distinct fibroblast subpopulations: ICAF_CXCL12, Fibroblast_LIMCH1 and myoCAF_FAP. (B) Marker genes expressed by these fibroblast subpopulations. (C and D) Changes in the proportions of fibroblast subpopulations in the lungs of IPF patients. (E) Single‐cell trajectory analysis suggests possible differentiation pathways among these three fibroblast subtypes.

Senescent fibroblasts are known to drive the progression of IPF [25]. In addition, metabolic abnormalities in fibroblasts and myofibroblasts in IPF lungs may contribute to excessive collagen synthesis and fibrosis [26]. Using scRNA‐seq, three fibroblast subtypes were identified: iCAF_CXCL12, fibroblast_LIMCH1 and myoCAF_FAP (Figure 2A). The iCAF_CXCL12 subtype expressed inflammation‐related genes such as IL6, IL33, CCL2 and CXCL12 (Figure 2B). While its proportion was elevated in IPF lungs, the increase was not statistically significant (Figure 2C,D). The myoCAF_FAP subtype expressed ACTA2, a myofibroblast marker (Figure 2B) and its proportion was significantly higher in the lungs of IPF patients compared to healthy controls (Figure 2C,D). In contrast, the fibroblast_LIMCH1 subtype, which expressed adhesion‐related molecules such as ITGA1 (Figure 2B), showed a significant reduction in its proportion in IPF lungs (Figure 2C,D). Trajectory analysis at the single‐cell level revealed that fibroblast_LIMCH1 could differentiate into both myoCAF_FAP and iCAF_CXCL12 in IPF lungs (Figure 2E). These findings align with the observed proportional changes among the fibroblast subpopulations, providing insights into fibroblast dynamics and their potential roles in the pathogenesis of IPF.

### 3.3 Single Cell RNA‐Seq Identifies Myeloid‐Derived Cells and Their Proportion Changes in the Lungs of IPF Patients

**FIGURE 3:**
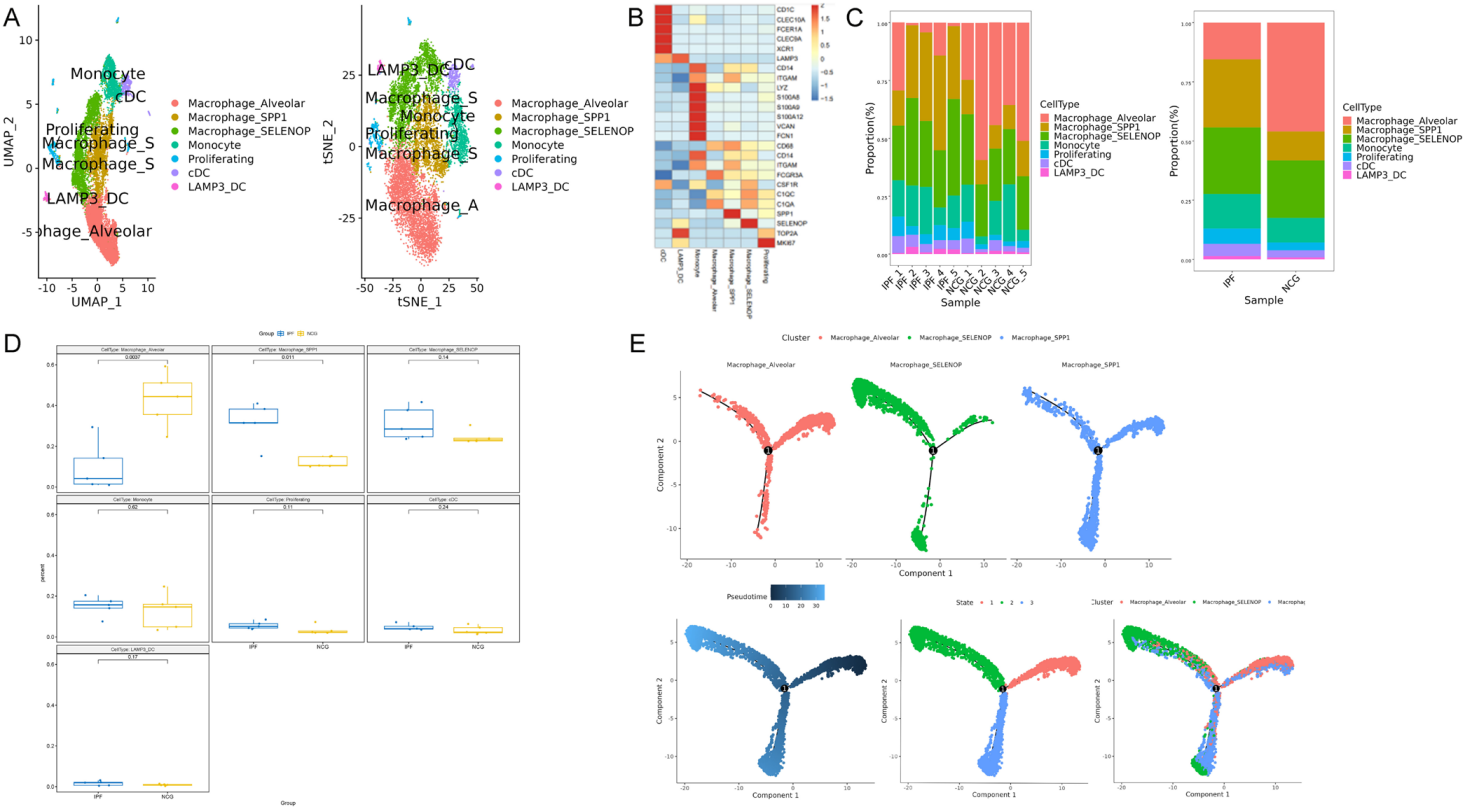
Single‐cell RNA‐seq identifies myeloid cell subpopulations and their altered proportions in the lungs of patients with idiopathic pulmonary fibrosis (IPF). Myeloid cells were extracted from the complete Seurat object and processed using the standard Seurat pipeline to identify and characterise these subpopulations. (A) The myeloid cells identified include macrophages, dendritic cells and monocytes. Among them, macrophages and dendritic cells are further subdivided into three and two distinct subpopulations respectively. (B) Marker genes expressed by the myeloid cells and their respective subpopulations. (C and D) Alterations in the proportions of myeloid cell populations and their subpopulations in the lungs of IPF patients compared to normal human lungs. (E) Single‐cell trajectory analysis reveals potential differentiation pathways between macrophage subpopulations.

Myeloid‐derived cells identified through scRNA‐seq included macrophages, monocytes and dendritic cells (Figure 3A,B). Among these, three distinct macrophage subpopulations and two dendritic cell subpopulations were identified (Figure 3A,B). Notably, a subset of cells expressing proliferation‐associated genes also exhibited dendritic cell marker gene expression (Figure 3A,B). The proportions of dendritic cell subpopulations did not significantly change between IPF patient lungs and normal human lungs (Figure 3C,D). Three subpopulations expressing the pan‐macrophage marker CD68 were identified, including the macrophage_Alveolar, macrophage_SPP1 and macrophage_SELENCP subpopulations, which were defined by markers ITGAX, SPP1 and SELENCP respectively (Figure 3C,D). The proportion of macrophage_Alveolar cells was significantly reduced in the lungs of IPF patients, while the proportions of macrophage_SPP1 and macrophage_SELENCP subpopulations were increased, although these changes were not statistically significant (Figure 3C,D). Single‐cell trajectory analysis revealed that macrophage_Alveolar cells could differentiate into macrophage_SPP1 and macrophage_SELENCP in IPF lungs (Figure 3E), providing further insight into macrophage dynamics and their potential role in IPF pathogenesis.

### 3.4 Single Cell RNA‐Seq Identifies B‐ and T‐Cell Subpopulations and Their Proportion Changes in the Lungs of IPF Patients

**FIGURE 4:**
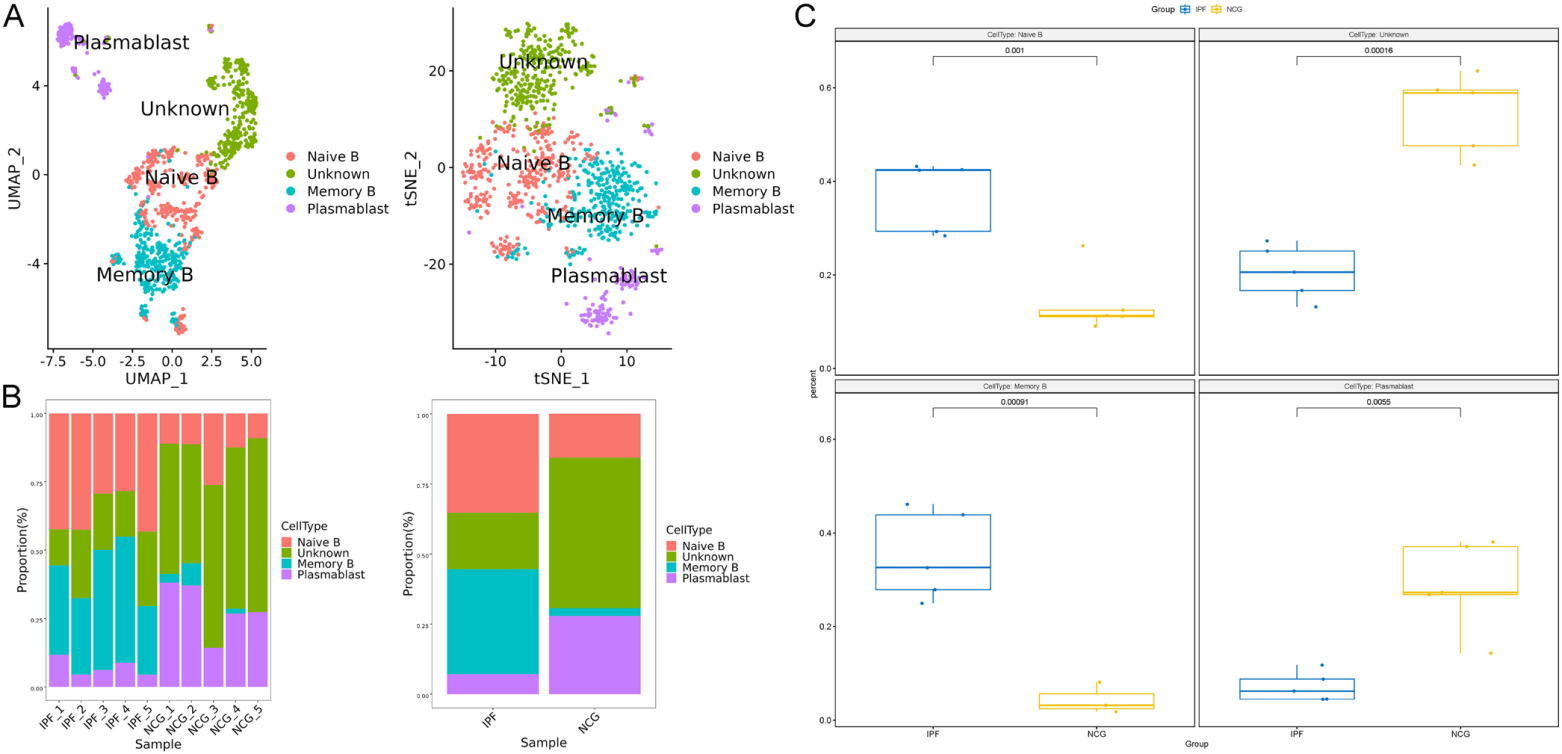
B‐cell subpopulations identified by single‐cell RNA‐seq and their alterations in the lungs of patients with idiopathic pulmonary fibrosis (IPF). B cells were extracted from the complete Seurat object and processed using the standard Seurat pipeline to identify and characterise B‐cell subpopulations. (A) Single‐cell RNA‐seq identified four distinct B‐cell subpopulations: Naïve B cells, memory B cells, plasmablasts and an undefined subpopulation. (B and C) Alterations in the proportions of B‐cell subpopulations in the lungs of IPF patients compared to normal human lungs.

ScRNA‐seq analysis identified four distinct B‐cell subpopulations: (1) Naive B cells, characterised by markers CD69, TXNIP, MS4A1, TYROBP and DUSP1; (2) Memory B cells, defined by markers LMNA, MARCKS, REL, EZR and KDM6B; (3) Plasmablasts, marked by GZMB, EGLN3, SLC7A5, ITM2C and APP and (4) Undefined B cells, characterised by markers PPP2R5C, YME1L1, ZFAND5, ARID4B and HNRNPL (Figure 4A). Among these subpopulations, the proportions of naive B cells and memory B cells were significantly increased in the lungs of IPF patients, while the proportions of undefined B cells and plasmablasts were significantly reduced (Figure 4B,C).

**FIGURE 5:**
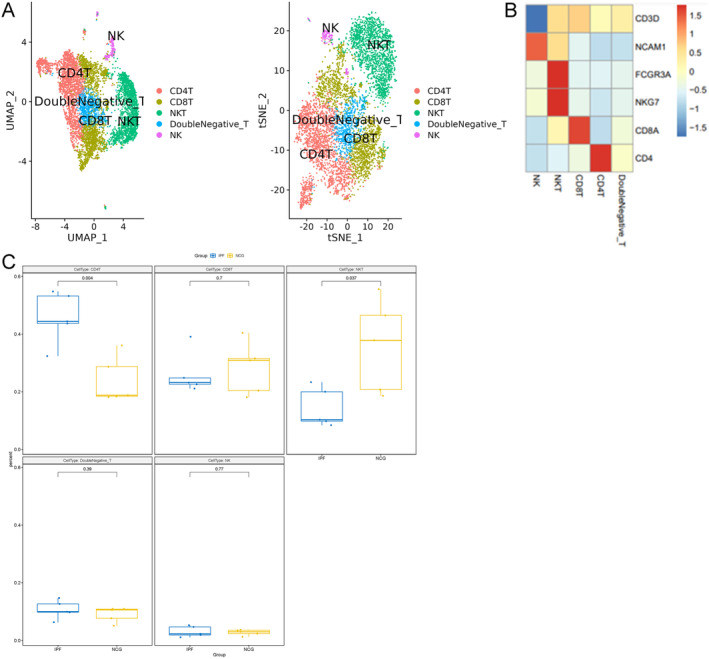
Four T‐cell subpopulations identified by single‐cell RNA‐seq and their alterations in the lungs of patients with idiopathic pulmonary fibrosis (IPF). T cells, NKT cells and NK cells were extracted from the complete Seurat object and processed using the standard Seurat pipeline. (A) The T‐cell populations identified by single‐cell RNA‐seq include CD4 T cells, CD8 T cells, double‐negative T cells and NKT cells. (B) The marker genes expressed by these T‐cell subpopulations. (C) Alterations in the proportions of T‐cell subpopulations in the lungs of IPF patients compared to normal human lungs.

In addition, scRNA‐seq identified four T‐cell types: CD4 T cells, CD8 T cells, double‐negative T cells and NKT cells (Figure 5A,B). The ratio of CD4 T cells was significantly increased in IPF lungs, while NKT cells were significantly reduced. However, the proportions of CD8 T cells and double‐negative T cells did not significantly differ between IPF patients and normal controls (Figure 5C).

**FIGURE 6:**
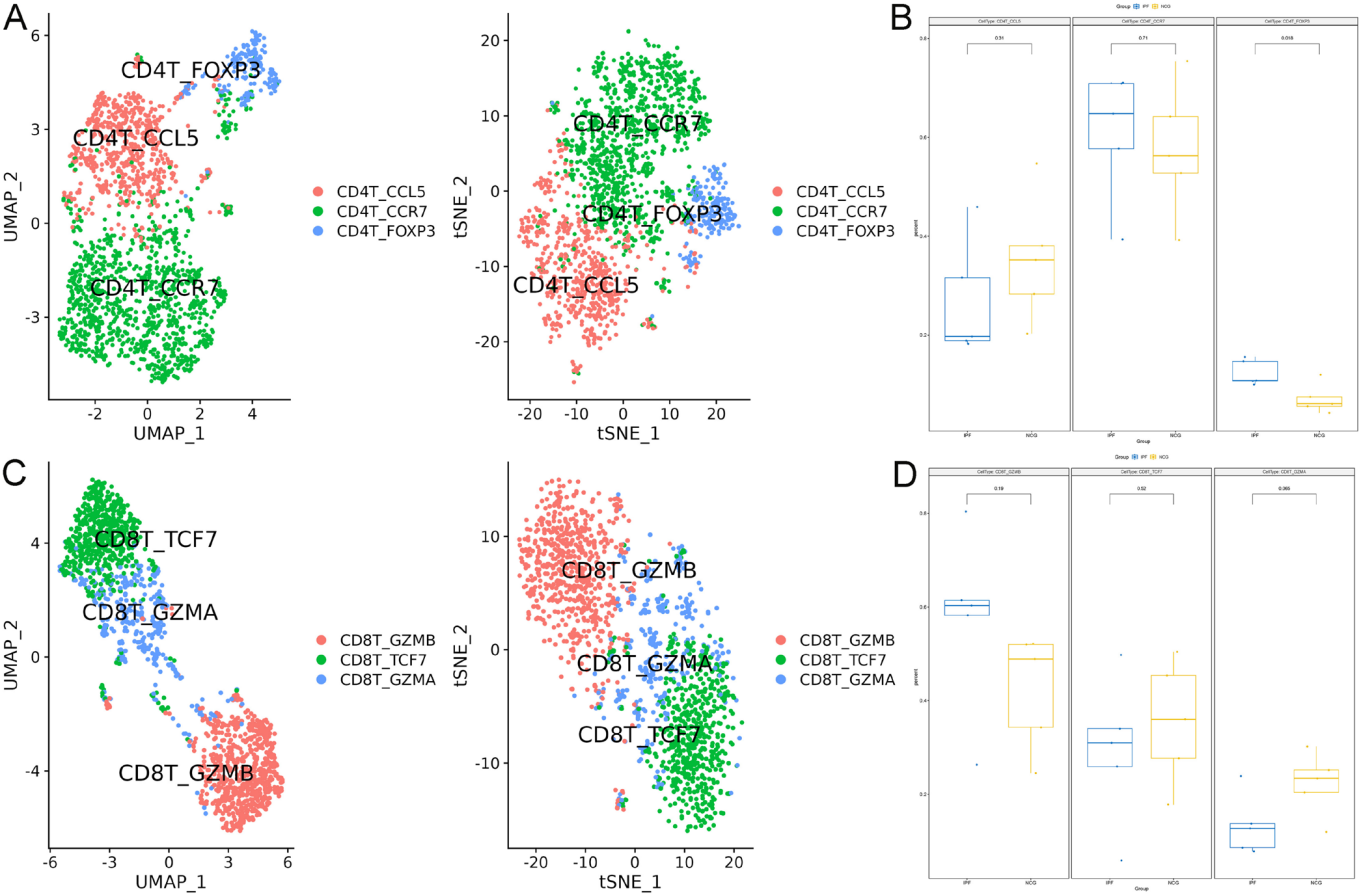
CD4 and CD8 T‐cell subpopulations identified by single‐cell RNA‐seq and their alterations in the lungs of patients with idiopathic pulmonary fibrosis (IPF). CD4 and CD8 T cells were extracted from the complete Seurat object and processed using the standard Seurat pipeline to identify and characterise their subpopulations. (A and C) CD4 T cells and CD8 T cells can be divided into three subpopulations: CD4_CCL5, CD4_CCR7 and CD4_FOXP3 for CD4 T cells, and CD8_GZMB, CD8_TCF7 and CD8_GZMA for CD8 T cells. (B and D) Alterations in the proportions of CD4 T‐cell and CD8 T‐cell subpopulations in the lungs of IPF patients compared to normal human lungs.

Further analysis of CD4 T cells and CD8 T cells revealed that each subset could be divided into three distinct subpopulations (Figure 6A,C). Notably, the CD4_FOXP3 subpopulation, likely comprising regulatory T cells due to its expression of the FOXP3 marker, exhibited a significant increase in the lungs of IPF patients. In contrast, the proportions of the other subpopulations of both CD4 T cells and CD8 T cells remained unchanged (Figure 6B,D). These findings suggest alterations in T‐cell populations, particularly regulatory T cells, in the context of IPF.

### 3.5 Extensive Cell–Cell Interactions Exist Between Cells or Subpopulations

**FIGURE 7:**
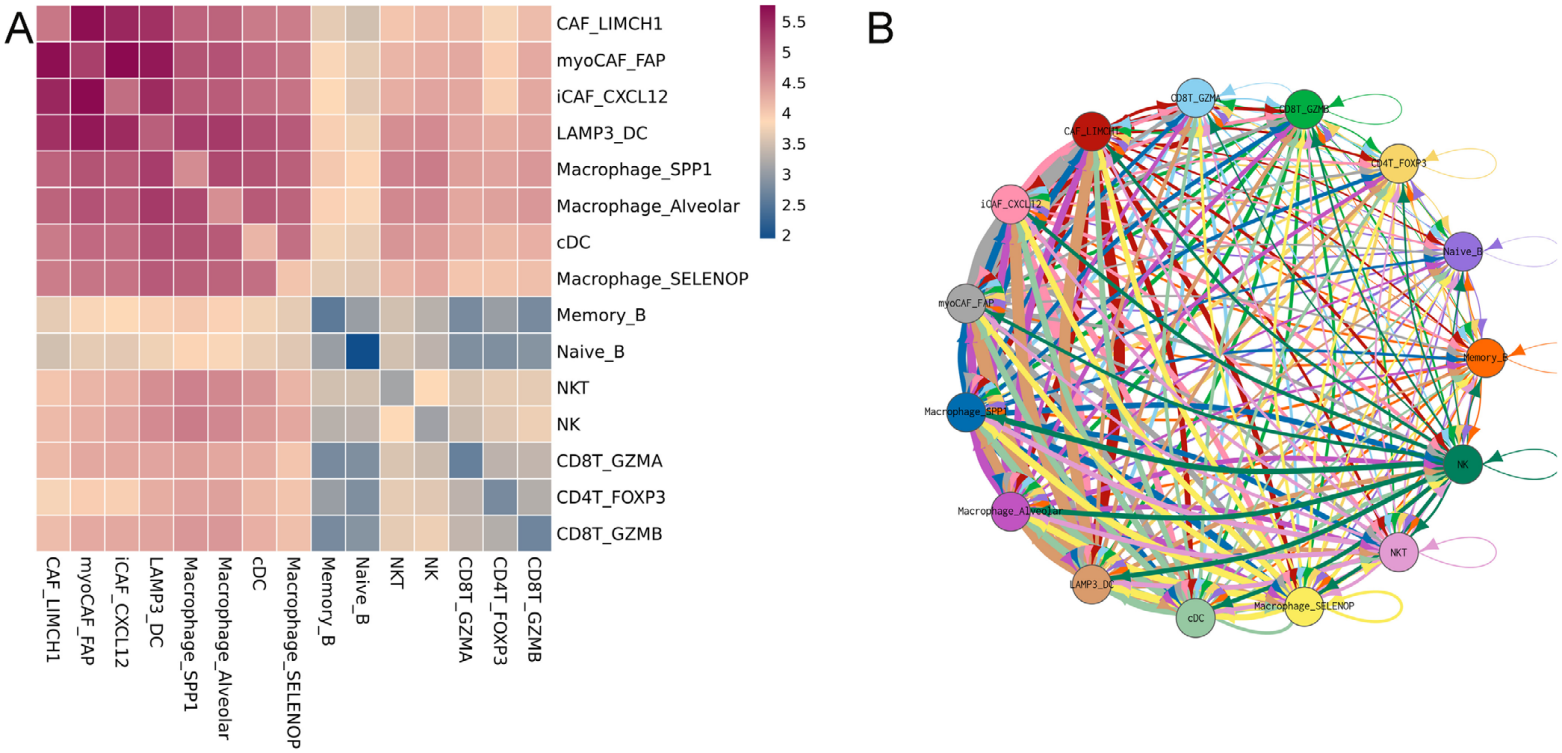
Extensive intercellular interactions occur between altered cell populations in the lungs of patients with idiopathic pulmonary fibrosis (IPF). These interactions were assessed using CellPhoneDB software. (A) The heatmap illustrates the extent of interactions between various cell populations or subpopulations. (B) The network diagram depicts the interactions between cells through receptors and their corresponding ligands.

Cell–cell interactions were analysed using CellphoneDB software to investigate the communication between different cell populations and subpopulations in IPF. Heatmaps were generated based on the number of interactions detected between these cell groups (Figure 7A). The analysis revealed that fibroblast–fibroblast interactions, macrophage–macrophage interactions and fibroblast–macrophage interactions were more prevalent, whereas T‐cell–T‐cell interactions, B‐cell–B‐cell interactions and T‐cell–B‐cell interactions were relatively fewer (Figure 7A). Receptors and their corresponding ligands were extracted and used to construct network diagrams, visualising the interactions among the various cell types (Figure 7B). Both fibroblasts and macrophages were found to engage in extensive interactions through receptors and ligands. Specifically, fibroblasts were involved in numerous interactions via growth factors, such as transforming growth factor (TGF), fibroblast growth factor (FGF) and platelet‐derived growth factor (PDGF), forming 30, 96 and 19 interactions, respectively, with other cell types and with fibroblasts themselves. Macrophages, on the other hand, produced a variety of cytokines, including tumour necrosis factor (TNF), chemokine (C‐X‐C motif) ligand (CXCL) and chemokine (C‐C motif) ligand (CCL), which formed 83, 33 and 83 interactions, respectively, with other cells and among macrophages.

These findings highlight the intricate network of signalling pathways involved in the pathogenesis of IPF, underscoring the complex interactions between various cell types that contribute to disease progression.

## 4 Discussion

IPF is a form of interstitial lung disease characterised by cellular proliferation, interstitial inflammation and progressive fibrosis [27]. Through single‐cell RNA sequencing, we identified a comprehensive array of relevant cell populations and subpopulations in IPF lungs, including endothelial cells, epithelial cells, fibroblasts, macrophages, T cells, B cells and others.

In IPF lungs, type 2 alveolar epithelial cells (AT2) exhibit increased apoptosis, senescence, abnormal differentiation and impaired renewal capacity [3, 28]. Consistent with these characteristics, our single‐cell RNA‐seq analysis revealed a reduction in the proportion of epithelial cells in IPF lungs. The senescence of AT2 cells is known to contribute significantly to the progression of pulmonary fibrosis [28]. In contrast, our analysis showed that the proportion of endothelial cells was elevated in IPF lungs compared to normal lungs. Vascular endothelial growth factor (VEGF) is upregulated in capillary endothelial cells in regions of the IPF lung spared from fibrosis [29], which helps inhibit endothelial cell apoptosis while promoting cell proliferation, migration and differentiation. These changes in the proportions of epithelial and endothelial cells suggest their potential involvement in the onset and progression of IPF.

Fibrosis is a hallmark of IPF, characterised by the excessive accumulation of extracellular matrix components, including collagen. Fibroblasts are the primary source of extracellular matrix in the lungs [30]. Our single‐cell RNA‐seq analysis identified three distinct fibroblast subpopulations: fibroblast_LIMCH1, iCAF_CXCL12, which express proinflammatory cytokines such as IL6, IL33, CCL2 and CXCL12 and myoCAF_FAP, which expresses the myofibroblast marker ACTA2. We found that the proportion of fibroblast_LIMCH1 was significantly reduced in IPF lungs, while the proportions of iCAF_CXCL12 and myoCAF_FAP were significantly increased. iCAF_CXCL12 fibroblasts play a key role in regulating inflammation in IPF lungs, which exhibit senescence features, such as telomere attrition and the expression of p21 and p16 [31, 32]. Myofibroblasts are strongly associated with increased collagen deposition [33] and, in IPF lungs, they may resist apoptosis due to the growth factor–rich environment, which includes platelet‐derived growth factor (PDGF) and vascular endothelial growth factor (VEGF) [3]. Additionally, fibroblasts with reduced ABHD5 expression have been shown to exhibit enhanced migratory ability and secrete higher levels of fibrotic factors [34]. Single‐cell trajectory analysis suggested that fibroblast_LIMCH1 may differentiate into iCAF_CXCL12 and myoCAF_ACTA2. In IPF lungs, the dynamic switching between these fibroblast subpopulations, leading to changes in their relative proportions and the generation of fibrosis‐promoting subpopulations, likely contributes to the progression of the disease.

In addition to cell proliferation and fibrosis, persistent chronic inflammation is a key characteristic of IPF [2, 3, 35]. Our single‐cell RNA‐seq analysis revealed significant changes in the proportions of several inflammation‐related cell populations or subpopulations in IPF lungs, including macrophages, T cells and B cells. Among the three macrophage subpopulations, macrophage_alveolar was significantly reduced, while the proportions of macrophage_SPP1 and macrophage_SELENCP were significantly increased in IPF lungs. Consistent with this, single‐cell trajectory analysis indicated that macrophage_alveolar can differentiate into the other two macrophage subpopulations. In contrast, the proportions of the two dendritic cell subpopulations did not change significantly in IPF lungs. Macrophage_SPP1 has been shown to localise in fibroblastic foci and exhibit an M2 phenotype [36], potentially playing a regulatory role in pulmonary fibrosis [15, 37]. SPP1 may influence lung fibrosis through its effects on anoikis and the PI3K/Akt signalling pathway [38]. These findings suggest that changes in macrophage populations, particularly the increased proportion of macrophage_SPP1, contribute to the inflammatory and fibrotic processes in IPF.

T cells have been well documented to play a role in regulating pulmonary fibrosis, but their precise involvement in the onset and progression of IPF remains to be fully understood [39, 40]. In our single‐cell RNA‐seq analysis, we identified three subpopulations of CD4 T cells and three subpopulations of CD8 T cells. However, their proportions did not show significant changes in IPF lungs, with the exception of the CD4 T_FOXP3 subpopulation. Notably, the CD4 T_FOXP3 population was found to be reduced in the peripheral blood and bronchoalveolar lavage of IPF patients [41]. This suggests that while T cells are implicated in IPF, specific subpopulations, particularly regulatory T cells (CD4 T_FOXP3), may be involved in the disease's pathogenesis.

In contrast to T cells, our single‐cell RNA‐seq analysis identified four distinct B‐cell subpopulations. Among these, two subpopulations showed a statistically significant increase, while the other two exhibited a significant decrease in IPF lungs. Naive B cells in IPF patients are known to exhibit abnormal B‐cell receptor (BCR) signalling [42], and their proportion is elevated in IPF lungs. Depletion of plasma B cells has been shown to reduce bleomycin‐induced pulmonary fibrosis in animal models of IPF [43, 44], yet our findings indicate that plasma B cells are significantly reduced in IPF lungs. Furthermore, B cells in IPF are implicated in enhancing Bruton's tyrosine kinase activity and may produce autoreactive IgA [45], while also promoting fibroblast migration and activation [45]. Additionally, our analysis uncovered a previously undefined B‐cell subpopulation. These observations underscore the potential role of B cells in the development and progression of IPF, suggesting that further investigation into their function and the targeting of B cells in therapeutic strategies may be warranted.

Our single‐cell RNA‐seq analysis revealed a broad array of potential cell–cell interactions in IPF. These interactions can occur through direct cell‐to‐cell contact or via the secretion of soluble factors that bind to specific receptors, thereby modulating cellular functions. Such interactions are known to promote fibroblast activation, differentiation and resistance to apoptosis [3]. In IPF lungs, cytokines such as IL‐13, IL‐4 and TGF‐β, secreted by T cells, are implicated in driving the differentiation of fibroblasts into myofibroblasts [46, 47]. These interactions also contribute to epithelial cell senescence and may induce NF‐κB activation, a pathway tightly linked to cellular senescence. Stimulation by various inflammatory and growth factors activates NF‐κB signalling, which is upregulated in IPF, and NF‐κB activation is essential for alveolar epithelial cell senescence [48]. Therefore, a deeper understanding of these intercellular interactions will not only enhance our comprehension of IPF pathogenesis but also inform the development of novel therapeutic strategies and drugs.

In summary, our single‐cell RNA‐seq analysis identified multiple cell subpopulations, with significant changes in their proportions in IPF lungs. These findings suggest that these subpopulations may play a critical role in the initiation and progression of IPF. This study not only enhances our understanding of the underlying pathology of IPF but also provides valuable insights for further investigation into the specific functions of these cell subpopulations.

## Author Contributions

**Tangjuan Zhang:** conceptualization (equal), data curation (equal), formal analysis (equal), writing – original draft (equal), writing – review and editing (equal). **Zhichao Hou:** data curation (equal), formal analysis (equal), writing – review and editing (equal). **Zheng Ding:** data curation (equal), formal analysis (equal), writing – review and editing (equal). **Peng Wang:** data curation (equal), formal analysis (equal), writing – review and editing (equal). **Xue Pan:** conceptualization (equal), data curation (equal), formal analysis (equal), writing – review and editing (equal). **Xiangnan Li:** conceptualization (equal), data curation (equal), formal analysis (equal), writing – review and editing (equal).

## Ethics Statement

This study was approved by the ethics committee of The First Affiliated Hospital of Zhengzhou University (No. 2019043). All procedures performed in studies involving human participants were in accordance with the ethical standards of the institutional and/or national research committee and with the 1964 Helsinki Declaration and its later amendments or comparable ethical standards.

## Consent

Written informed consent was obtained from individual participants.

## Conflicts of Interest

The authors declare no conflicts of interest.

## Supporting information


**Figure S1.** Representative lung CT images of patients with idiopathic pulmonary fibrosis (IPF). The figure presents four CT images for each IPF patient, highlighting the characteristic radiographic features commonly associated with IPF.

**Figure S2.** This flowchart illustrates the entire experimental procedure, starting from patient selection and sample preparation through to data analysis.

## Data Availability

The datasets used and/or analysed during the current study are available from the corresponding author on reasonable request.

## References

[1] D. J. Lederer and F. J. Martinez , “Idiopathic Pulmonary Fibrosis,” New England Journal of Medicine 379 (2018): 797–798. 10.1056/NEJMc1807508 30134133

[2] F. J. Martinez , H. R. Collard , A. Pardo , et al., “Idiopathic Pulmonary Fibrosis,” Nature Reviews. Disease Primers 3 (2017): 17074. 10.1038/nrdp.2017.74 29052582

[3] B. J. Moss , S. W. Ryter , and I. O. Rosas , “Pathogenic Mechanisms Underlying Idiopathic Pulmonary Fibrosis,” Annual Review of Pathology 17 (2022): 515–546. 10.1146/annurev-pathol-042320-030240 34813355

[4] L. Richeldi , H. R. Collard , and M. G. Jones , “Idiopathic Pulmonary Fibrosis,” Lancet 389 (2017): 1941–1952. 28365056 10.1016/S0140-6736(17)30866-8

[5] P. Spagnolo , J. A. Kropski , M. G. Jones , et al., “Idiopathic Pulmonary Fibrosis: Disease Mechanisms and Drug Development,” Pharmacology & Therapeutics 222 (2021): 107798. 33359599 10.1016/j.pharmthera.2020.107798 PMC8142468

[6] B. Ley , H. R. Collard , and T. E. King, Jr. , “Clinical Course and Prediction of Survival in Idiopathic Pulmonary Fibrosis,” American Journal of Respiratory and Critical Care Medicine 183 (2011): 431–440. 20935110 10.1164/rccm.201006-0894CI

[7] J. Hutchinson , A. Fogarty , R. Hubbard , and T. McKeever , “Global Incidence and Mortality of Idiopathic Pulmonary Fibrosis: A Systematic Review,” European Respiratory Journal 46 (2015): 795–806. 25976683 10.1183/09031936.00185114

[8] D. B. Esposito , S. Lanes , M. Donneyong , et al., “Idiopathic Pulmonary Fibrosis in United States Automated Claims. Incidence, Prevalence, and Algorithm Validation,” American Journal of Respiratory and Critical Care Medicine 192 (2015): 1200–1207. 26241562 10.1164/rccm.201504-0818OC

[9] M. Natsuizaka , H. Chiba , K. Kuronuma , et al., “Epidemiologic Survey of Japanese Patients With Idiopathic Pulmonary Fibrosis and Investigation of Ethnic Differences,” American Journal of Respiratory and Critical Care Medicine 190 (2014): 773–779. 25162152 10.1164/rccm.201403-0566OC

[10] G. Raghu , S. Y. Chen , Q. Hou , W. S. Yeh , and H. R. Collard , “Incidence and Prevalence of Idiopathic Pulmonary Fibrosis in US Adults 18‐64 Years Old,” European Respiratory Journal 48 (2016): 179–186. 27126689 10.1183/13993003.01653-2015

[11] G. Raghu , S. Y. Chen , W. S. Yeh , et al., “Idiopathic Pulmonary Fibrosis in US Medicare Beneficiaries Aged 65 Years and Older: Incidence, Prevalence, and Survival, 2001–11,” Lancet Respiratory Medicine 2 (2014): 566–572. 24875841 10.1016/S2213-2600(14)70101-8

[12] G. Raghu , B. Rochwerg , Y. Zhang , et al., “An Official ATS/ERS/JRS/ALAT Clinical Practice Guideline: Treatment of Idiopathic Pulmonary Fibrosis. An Update of the 2011 Clinical Practice Guideline,” American Journal of Respiratory and Critical Care Medicine 192 (2015): e3–e19. 26177183 10.1164/rccm.201506-1063ST

[13] B. Hwang , J. H. Lee , and D. Bang , “Single‐Cell RNA Sequencing Technologies and Bioinformatics Pipelines,” Experimental & Molecular Medicine 50 (2018): 1–14. 10.1038/s12276-018-0071-8 PMC6082860 30089861

[14] D. Jovic , X. Liang , H. Zeng , L. Lin , F. Xu , and Y. Luo , “Single‐Cell RNA Sequencing Technologies and Applications: A Brief Overview,” Clinical and Translational Medicine 12 (2022): e694. 35352511 10.1002/ctm2.694 PMC8964935

[15] P. A. Reyfman , J. M. Walter , N. Joshi , et al., “Single‐Cell Transcriptomic Analysis of Human Lung Provides Insights Into the Pathobiology of Pulmonary Fibrosis,” American Journal of Respiratory and Critical Care Medicine 199 (2019): 1517–1536. 30554520 10.1164/rccm.201712-2410OC PMC6580683

[16] T. S. Adams , J. C. Schupp , S. Poli , et al., “Single‐Cell RNA‐Seq Reveals Ectopic and Aberrant Lung‐Resident Cell Populations in Idiopathic Pulmonary Fibrosis,” Science Advances 6 (2020): eaba1983. 32832599 10.1126/sciadv.aba1983 PMC7439502

[17] A. C. Habermann , A. J. Gutierrez , L. T. Bui , et al., “Single‐Cell RNA Sequencing Reveals Profibrotic Roles of Distinct Epithelial and Mesenchymal Lineages in Pulmonary Fibrosis,” Science Advances 6 (2020): eaba1972. 32832598 10.1126/sciadv.aba1972 PMC7439444

[18] T. Stuart , A. Butler , P. Hoffman , et al., “Comprehensive Integration of Single‐Cell Data,” Cell 177 (2019): 1888–1902.e21. 31178118 10.1016/j.cell.2019.05.031 PMC6687398

[19] A. Butler , P. Hoffman , P. Smibert , E. Papalexi , and R. Satija , “Integrating Single‐Cell Transcriptomic Data Across Different Conditions, Technologies, and Species,” Nature Biotechnology 36 (2018): 411–420. 10.1038/nbt.4096 PMC6700744 29608179

[20] Y. Hao , T. Stuart , M. H. Kowalski , et al., “Dictionary Learning for Integrative, Multimodal and Scalable Single‐Cell Analysis,” Nature Biotechnology 42 (2023): 293–304. 10.1038/s41587-023-01767-y PMC10928517 37231261

[21] M. Efremova , M. Vento‐Tormo , S. A. Teichmann , and R. Vento‐Tormo , “CellPhoneDB: Inferring Cell‐Cell Communication From Combined Expression of Multi‐Subunit Ligand‐Receptor Complexes,” Nature Protocols 15 (2020): 1484–1506. 32103204 10.1038/s41596-020-0292-x

[22] R. Vento‐Tormo , M. Efremova , R. A. Botting , et al., “Single‐Cell Reconstruction of the Early Maternal‐Fetal Interface in Humans,” Nature 563 (2018): 347–353. 30429548 10.1038/s41586-018-0698-6 PMC7612850

[23] C. Trapnell , D. Cacchiarelli , J. Grimsby , et al., “The Dynamics and Regulators of Cell Fate Decisions Are Revealed by Pseudotemporal Ordering of Single Cells,” Nature Biotechnology 32 (2014): 381–386. 10.1038/nbt.2859 PMC4122333 24658644

[24] X. Qiu , Q. Mao , Y. Tang , et al., “Reversed Graph Embedding Resolves Complex Single‐Cell Trajectories,” Nature Methods 14 (2017): 979–982. 28825705 10.1038/nmeth.4402 PMC5764547

[25] D. W. Waters , K. E. C. Blokland , P. S. Pathinayake , et al., “Fibroblast Senescence in the Pathology of Idiopathic Pulmonary Fibrosis,” American Journal of Physiology. Lung Cellular and Molecular Physiology 315 (2018): L162–L172. 29696986 10.1152/ajplung.00037.2018 PMC6139657

[26] W. Roque and F. Romero , “Cellular Metabolomics of Pulmonary Fibrosis, From Amino Acids to Lipids,” American Journal of Physiology‐Cell Physiology 320 (2021): C689–C695. 33471621 10.1152/ajpcell.00586.2020 PMC8163573

[27] D. J. Lederer and F. J. Martinez , “Idiopathic Pulmonary Fibrosis,” New England Journal of Medicine 378 (2018): 1811–1823. 29742380 10.1056/NEJMra1705751

[28] C. Yao , X. Guan , G. Carraro , et al., “Senescence of Alveolar Type 2 Cells Drives Progressive Pulmonary Fibrosis,” American Journal of Respiratory and Critical Care Medicine 203 (2021): 707–717. 32991815 10.1164/rccm.202004-1274OC PMC7958503

[29] G. P. Cosgrove , K. K. Brown , W. P. Schiemann , et al., “Pigment Epithelium‐Derived Factor in Idiopathic Pulmonary Fibrosis: A Role in Aberrant Angiogenesis,” American Journal of Respiratory and Critical Care Medicine 170 (2004): 242–251. 15117744 10.1164/rccm.200308-1151OC

[30] N. W. Todd , I. G. Luzina , and S. P. Atamas , “Molecular and Cellular Mechanisms of Pulmonary Fibrosis,” Fibrogenesis & Tissue Repair 5 (2012): 11. 22824096 10.1186/1755-1536-5-11 PMC3443459

[31] D. Alvarez , N. Cardenes , J. Sellares , et al., “IPF Lung Fibroblasts Have a Senescent Phenotype,” American Journal of Physiology. Lung Cellular and Molecular Physiology 313 (2017): L1164–L1173. 28860144 10.1152/ajplung.00220.2017 PMC6148001

[32] M. J. Schafer , T. A. White , K. Iijima , et al., “Cellular Senescence Mediates Fibrotic Pulmonary Disease,” Nature Communications 8 (2017): 14532. 10.1038/ncomms14532 PMC5331226 28230051

[33] J. J. Tomasek , G. Gabbiani , B. Hinz , C. Chaponnier , and R. A. Brown , “Myofibroblasts and Mechano‐Regulation of Connective Tissue Remodelling,” Nature Reviews. Molecular Cell Biology 3 (2002): 349–363. 11988769 10.1038/nrm809

[34] Y. Liao , X. Peng , Y. Yang , et al., “Exploring ABHD5 as a Lipid‐Related Biomarker in Idiopathic Pulmonary Fibrosis: Integrating Machine Learning, Bioinformatics, and In Vitro Experiments,” Inflammation (2024), 10.1007/s10753-024-02107-1. 39046603

[35] K. Shenderov , S. L. Collins , J. D. Powell , and M. R. Horton , “Immune Dysregulation as a Driver of Idiopathic Pulmonary Fibrosis,” Journal of Clinical Investigation 131 (2021): e143226. 33463535 10.1172/JCI143226 PMC7810481

[36] Y. Liao , X. Peng , Y. Yang , et al., “Integrating Cellular Experiments, Single‐Cell Sequencing, and Machine Learning to Identify Endoplasmic Reticulum Stress Biomarkers in Idiopathic Pulmonary Fibrosis,” Annals of Medicine 56 (2024): 2409352. 39340293 10.1080/07853890.2024.2409352 PMC11441044

[37] T. Tsukui , K. H. Sun , J. B. Wetter , et al., “Collagen‐Producing Lung Cell Atlas Identifies Multiple Subsets With Distinct Localization and Relevance to Fibrosis,” Nature Communications 11 (2020): 1920. 10.1038/s41467-020-15647-5 PMC7174390 32317643

[38] Y. Liao , Y. Yang , G. Zhou , et al., “Anoikis and SPP1 in Idiopathic Pulmonary Fibrosis: Integrating Bioinformatics, Cell, and Animal Studies to Explore Prognostic Biomarkers and PI3K/AKT Signaling Regulation,” Expert Review of Clinical Immunology 20 (2024): 679–693. 38318669 10.1080/1744666X.2024.2315218

[39] S. Kolahian , I. E. Fernandez , O. Eickelberg , and D. Hartl , “Immune Mechanisms in Pulmonary Fibrosis,” American Journal of Respiratory Cell and Molecular Biology 55 (2016): 309–322. 27149613 10.1165/rcmb.2016-0121TR

[40] G. J. Nuovo , J. S. Hagood , C. M. Magro , et al., “The Distribution of Immunomodulatory Cells in the Lungs of Patients With Idiopathic Pulmonary Fibrosis,” Modern Pathology 25 (2012): 416–433. 22037258 10.1038/modpathol.2011.166 PMC3270219

[41] I. Kotsianidis , E. Nakou , I. Bouchliou , et al., “Global Impairment of CD4+CD25+FOXP3+ Regulatory T Cells in Idiopathic Pulmonary Fibrosis,” American Journal of Respiratory and Critical Care Medicine 179 (2009): 1121–1130. 19342412 10.1164/rccm.200812-1936OC

[42] S. F. H. Neys , P. Heukels , J. A. C. van Hulst , et al., “Aberrant B Cell Receptor Signaling in Naive B Cells From Patients With Idiopathic Pulmonary Fibrosis,” Cells 10 (2021): 1321. 34073225 10.3390/cells10061321 PMC8226954

[43] A. T. Goodwin , P. W. Noble , and A. L. Tatler , “Plasma Cells: A Feasible Therapeutic Target in Pulmonary Fibrosis?,” European Respiratory Journal 60 (2022): 2201748. 36423920 10.1183/13993003.01748-2022

[44] C. M. Prele , T. Miles , D. R. Pearce , et al., “Plasma Cell but Not CD20‐Mediated B‐Cell Depletion Protects From Bleomycin‐Induced Lung Fibrosis,” European Respiratory Journal 60 (2022): 2101469. 35798357 10.1183/13993003.01469-2021 PMC9684624

[45] M. F. Ali , A. M. Egan , G. F. Shaughnessy , et al., “Antifibrotics Modify B‐Cell‐Induced Fibroblast Migration and Activation in Patients With Idiopathic Pulmonary Fibrosis,” American Journal of Respiratory Cell and Molecular Biology 64 (2021): 722–733. 33689587 10.1165/rcmb.2020-0387OC PMC8456878

[46] S. Hashimoto , Y. Gon , I. Takeshita , S. Maruoka , and T. Horie , “IL‐4 and IL‐13 Induce Myofibroblastic Phenotype of Human Lung Fibroblasts Through c‐Jun NH2‐Terminal Kinase‐Dependent Pathway,” Journal of Allergy and Clinical Immunology 107 (2001): 1001–1008. 11398077 10.1067/mai.2001.114702

[47] L. J. Celada , J. A. Kropski , J. D. Herazo‐Maya , et al., “PD‐1 Up‐Regulation on CD4(+) T Cells Promotes Pulmonary Fibrosis Through STAT3‐Mediated IL‐17A and TGF‐beta1 Production,” Science Translational Medicine 10 (2018): eaar8356. 30257954 10.1126/scitranslmed.aar8356 PMC6263177

[48] Y. Tian , H. Li , T. Qiu , et al., “Loss of PTEN Induces Lung Fibrosis via Alveolar Epithelial Cell Senescence Depending on NF‐kappaB Activation,” Aging Cell 18 (2019): e12858. 30548445 10.1111/acel.12858 PMC6351835

