# Supplementary figures and images for: Single Cell RNA‐Seq Identifies Cell Subpopulations Contributing to Idiopathic Pulmonary Fibrosis in Humans
Source: J Cell Mol Med. 2025 Feb 10;29(3):e70402. doi: 10.1111/jcmm.70402 (PMC11809556; doi:10.1111/jcmm.70402)

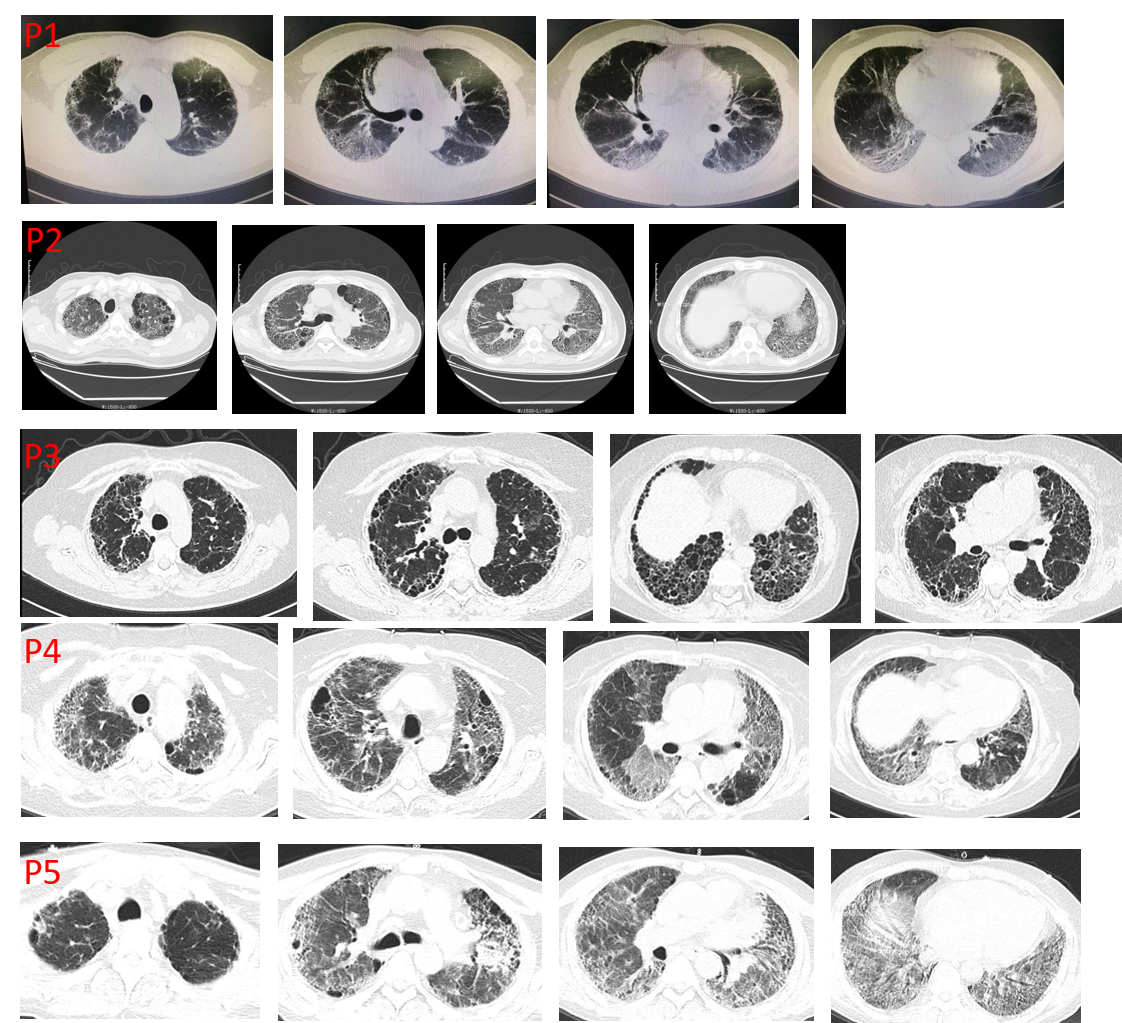

Supplement: Supplementary file 1 — Figure S1. Representative lung CT images of patients with idiopathic pulmonary fibrosis (IPF). The figure presents four CT images for each IPF patient, highlighting the characteristic radiographic features commonly associated with IPF. [file JCMM-29-e70402-s002.tif]

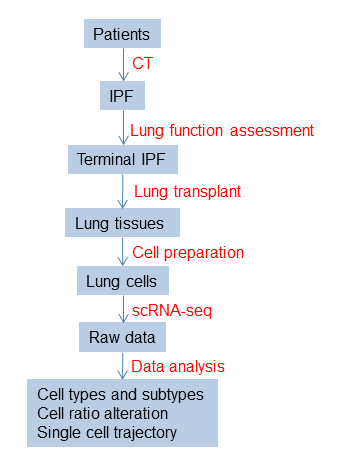

Supplement: Supplementary file 2 — Figure S2. This flowchart illustrates the entire experimental procedure, starting from patient selection and sample preparation through to data analysis. [file JCMM-29-e70402-s001.tif]
